# Four distinct peer interaction variables as moderators of the fearful temperament‐anxiety association, using data from the Generation R Study

**DOI:** 10.1002/jcv2.12254

**Published:** 2024-06-19

**Authors:** Anita Harrewijn, Rosa H. Mulder, Marinus H. van IJzendoorn, Matthias J. Wieser, Pauline W. Jansen

**Affiliations:** ^1^ Department of Psychology, Education and Child Studies Erasmus University Rotterdam Rotterdam the Netherlands; ^2^ Department of Child and Adolescent Psychiatry/Psychology Erasmus MC University Medical Center Rotterdam Rotterdam the Netherlands; ^3^ The Generation R Study Group Erasmus MC University Medical Center Rotterdam Rotterdam the Netherlands; ^4^ Department of Psychiatry Monash University Clayton Victoria Australia; ^5^ Research Department of Clinical Education and Health Psychology University College London London UK

**Keywords:** adolescence, anxiety, fearful temperament, peer interactions, social exclusion

## Abstract

**Background:**

Pediatric anxiety disorders are common and have severe long‐term consequences. Early‐life fearful temperament is a predictor of later anxiety, but not all children with highly fearful temperament will eventually develop an anxiety disorder. Therefore, it is important to identify factors that moderate the fearful temperament‐anxiety association. The goal of this study it to replicate the fearful temperament‐anxiety association in a large cohort study, explore sex as a moderator of this association, and to investigate four distinct peer interaction variables as moderators of this association.

**Methods:**

2730 children (51.0% girls) with parent‐reported fearful temperament at 6 months and parent‐reported anxiety symptoms at 13 years were included from a prospective cohort study (Generation R Study). Fearful temperament was also observed in a subset (*n* = 643, 49.3% girls) of these children. Peer interactions were measured in four different ways: mother‐reported victimization (at age 7), self‐reported friendship quality (at age 9), and self‐reported feelings and facial expressions during social exclusion in a lab‐based task (at age 9).

**Results:**

Children with higher parent‐reported, but not observed, fearful temperament showed more anxiety symptoms as adolescents, *β* = 0.07, *p* < 0.001. This association was not moderated by sex, *β* = −0.07, *p* = 0.07, but was stronger in adolescents who reported more negative feelings after social exclusion, *β* = 0.05, *p* = 0.04. Victimization, friendship quality, and sad facial expressions were related to increased anxiety symptoms but did not moderate the fearful temperament‐anxiety association.

**Conclusions:**

We showed that parent‐reported fearful temperament and anxiety were associated in this large community sample and that this association was not moderated by sex. Additionally, we showed that negative feelings after social exclusion moderated this association. Potentially, children with a highly fearful temperament might benefit from learning how to cope with social exclusion. Future studies are needed to confirm our findings and could focus on the potential role of coping with social rejection in interventions.

## Graphical abstract

The goal of this study was to replicate the fearful temperament‐anxiety association in a large cohort study, and to investigate four distinct peer interaction variales as moderators of this association. We showed that parent‐reported temperament and anxiety were associated in this large community sample (*n* = 2730). This association was stronger in adolescents who reported more negative feelings after social exclusion.

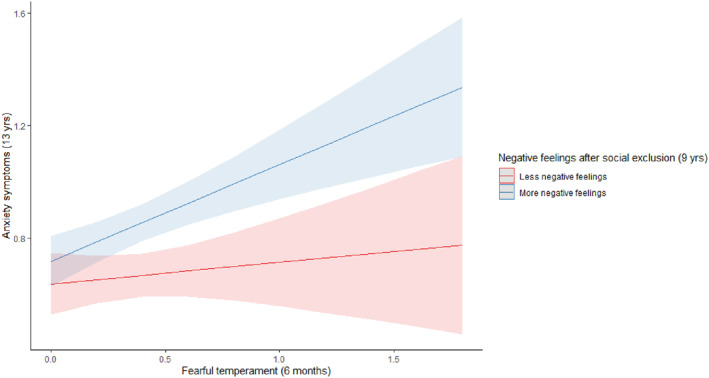

**Key points**

- We studied the temperament‐anxiety association, and sex and four distinct peer interaction variables as potential moderators of this association in a large prospective cohort study.
- Parent‐reported fearful temperament at 6 months was associated with anxiety symptoms at 13 years.
- This association was stronger in adolescents who reported more negative feelings after social exclusion.
- Future studies are needed to confirm our findings and could focus on the potential role of coping with social rejection in interventions.

## INTRODUCTION

Pediatric anxiety disorders are common and have severe consequences (Copeland et al., 2014; Costello et al., 2005; Essau et al., 2000; Ezpeleta et al., 2001; Merikangas et al., 2010). Early‐life highly fearful temperament is a strong predictor of later anxiety (Clauss & Blackford, 2012; Sandstrom et al., 2020). However, not all children with a highly fearful temperament will eventually develop an anxiety disorder. Therefore, it is important to unravel other factors that influence the fearful temperament‐anxiety association to improve early detection and prevention. Most studies have focused on parental factors, but peer factors are also very important during childhood and adolescence (Giletta et al., 2021). Therefore, we will focus on how fearful temperament and peer interactions together predict later anxiety. The goal of this study it to replicate the fearful temperament‐anxiety association in a large cohort study, and to investigate four distinct peer interaction variables as moderators of the fearful temperament‐anxiety association.

Early‐life fearful temperament is characterized by fear and avoidance of novelty (Fox et al., 2005; Garcia Coll et al., 1984; Kagan et al., 1984), it is a broader construct encompassing different dimensions of temperament (e.g., behavioral inhibition, social reticence) (Fox et al., 2023). Many studies have shown that early‐life highly fearful temperament is related to later anxiety in childhood, adolescence, and emerging adulthood (Clauss & Blackford, 2012; Garcia‐Lopez et al., 2020; Hudson et al., 2019; Rapee, 2014; Sandstrom et al., 2020). Even decades later, highly fearful temperament was found to predict anxiety (Frenkel et al., 2015), depression (Caspi et al., 1996), and reserved personality (Tang et al., 2020). These findings were robust across different study designs with fearful temperament being studied in different ways, using parent‐reported questionnaires and/or behavioral observations and focusing on reactions to social and/or non‐social situations (Clauss et al., 2015; Fox et al., 2023). Even though anxiety is more prevalent in females than males (Kessler et al., 2005), the fearful temperament‐anxiety association does not seem to be moderated by sex (Hudson et al., 2019; Sandstrom et al., 2020). However, the association between shyness and social anxiety was stronger in girls than boys (Tsui et al., 2017), suggesting the need for more research on sex‐specificity. The *first goal* of this study is to replicate the fearful temperament‐anxiety association in a large cohort study and to explore sex as a moderator of this association.

Of all children, 15%–20% exhibit a highly fearful temperament in early childhood (Fox et al., 2005), and the risk of developing anxiety is almost three times higher in these children with highly fearful temperament (Sandstrom et al., 2020). Identifying moderators of the fearful temperament‐anxiety association could improve early detection and prevention of anxiety. Previous research has shown that internal factors such as increased detection of novelty or threat (e.g. attention bias to threat, error processing) and automatic control increase the risk of anxiety in children with fearful temperament, whereas planful control (e.g. proactive control) decreases the risk for anxiety (Fox et al., 2021, 2023; Liu & Perez‐Edgar, 2019). Most research on external factors has focused on parental factors, such as parental control behaviors (e.g. overcontrol and overprotection), parenting/parental stress, and attachment (Essex et al., 2010; Fox et al., 2023; Goldsmith et al., 2022; Hudson et al., 2019; Liu & Perez‐Edgar, 2019). However, interactions with peers are very important as well and peer influence effects have been found on a range of behaviors, including internalizing symptoms (Giletta et al., 2021). Thus, peer interactions in childhood might influence which children with a highly fearful temperament are at increased risk for developing anxiety.

Peer interactions are studied in many ways, varying in scope, developmental timing, and informant. In children with a highly fearful temperament, their risk for anxiety is increased when these children have experienced peer rejection, exclusion, victimization, and poor friendship quality (Degnan et al., 2010). However, another study found no interaction between fearful temperament and peer victimization in predicting internalizing symptoms, only in externalizing symptoms (Boyd et al., 2022). Another moderator of the fearful temperament‐anxiety association is social involvement: fearful temperament was related to adult anxiety when these children showed low involvement in social peer networks during adolescence (Frenkel et al., 2015). So, only few studies have investigated peer interactions as potential moderators of the fearful temperament‐anxiety association, relying on relatively small samples with questionnaire data only. Therefore, we aim to study several peer interaction variables as moderators of the fearful‐temperament‐anxiety association in a large cohort study.

Here we will focus on four distinct measures of peer interactions that were included in the Generation R Study, to give a broad overview in scope, timing, and informant. The first measure of peer interactions is victimization, which is a risk factor for anxiety (Pickering et al., 2020; Pontillo et al., 2019; Stapinski et al., 2014) and a moderator (Degnan et al., 2010) and mediator (Affrunti et al., 2014) of the fearful temperament‐anxiety association. The second measure is friendship quality, which seems to be mostly related to social anxiety (Chiu et al., 2021; Pickering et al., 2020), but also improves treatment outcome in children with any anxiety disorder (Baker & Hudson, 2013). Friendship quality is also a potential moderator of the fearful temperament‐anxiety association, but the direction of the association is unclear and potentially dependent on the (fearful) temperament of the friend (Degnan et al., 2010). The third measure is negative feelings during or after social exclusion as measured with the Cyberball paradigm, in which participants are excluded during a virtual ball‐tossing game. Ostracism distress is related to anxiety sensitivity in children (Davidson et al., 2019). The fourth measure is facial expressions during the Cyberball task, which is a novel measure that has not yet been studied in relation to anxiety or temperament, but is negatively related to later internalizing problems in the same sample (Mulder et al., 2023). The *second goal* of this study is to extend previous research by focusing on victimization, friendship quality, and responses to social exclusion as potential moderators of the fearful temperament‐anxiety association in a large cohort study using a multi‐modal approach.

The goals of the current, pre‐registered study are twofold: 1) Replicate the fearful temperament‐anxiety association in a large cohort study and explore sex as a moderator of this association; 2) Investigate different measures of peer interactions as moderators of the fearful temperament‐anxiety association (Harrewijn et al., 2021). We used data from the Generation R Study, which is a large prospective cohort study. Fearful temperament was measured with a parent‐reported questionnaire when infants were 6 months and with behavioral observations in a subset of the children when they were 3 years. We included multi‐modal assessment of peer interactions: mother‐reported victimization (at age 7), self‐reported friendship quality (at age 9), and self‐reported feelings and facial expressions during social exclusion in a lab‐based task (at age 9). First, as described in our pre‐registration (Harrewijn et al., 2021), we hypothesized that children with higher levels of early‐life fearful temperament would show more anxiety symptoms in adolescence (Clauss & Blackford, 2012; Fox et al., 2005; Sandstrom et al., 2020). We had no hypothesis about the direction of this moderation by sex. Second, we hypothesized that children with higher levels of early‐life fearful temperament would show more anxiety symptoms in adolescence especially if they also experienced more victimization, less friendship quality, and more self‐reported negative feelings during/after social exclusion, and displayed more negative facial expressions during social exclusion (Degnan et al., 2010; Frenkel et al., 2015).

## METHODS

### Participants

This study was part of the Generation R Study in Rotterdam, The Netherlands, which is a prospective cohort study. The general goal of the Generation R Study is to investigate environmental and genetic influences on normal and abnormal development and health (Kooijman et al., 2016). The study started with 9778 mothers who were followed since pregnancy, with assessments at home or in the research center during pregnancy and the preschool, mid childhood, and adolescence periods. Eligibility criteria were residence of a designated postal code area in Rotterdam and expected delivery date between April 2002 and January 2006 (Kooijman et al., 2016). At birth, 9749 children were enrolled. Study procedures were approved by the Medical Ethics Committee of the Erasmus University Medical Center. Parents provided informed consent for their children, and children aged 12 years and older also signed their own consent form, in accordance with Dutch law.

**FIGURE 1:**
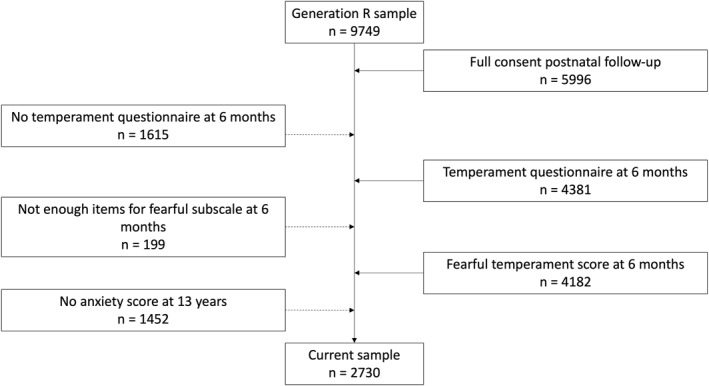
Flow‐chart depicting inclusion in the current study.

The current study included children with data on fearful temperament at 6 months and anxiety at 13 years. Parents of 4182 children reported on fearful temperament at 6 months, of whom 2730 children (51.0% girls) also had data on parent‐reported anxiety at 13 years (Figure 1, Table 1, Table S1). Of those 2730 children, fearful temperament was assessed through observations in a subset of 643 children (49.3% girls) children at 3 years. Of the final sample with parent‐reported fearful temperament, 1913 children (70.1%) were Dutch and 808 (29.6%) were not Dutch, information on national origin was missing for 9 children (0.3%). 1666 mothers (61.0%) completed university or university of applied sciences and 981 mothers (35.9%) completed other education (secondary education, vocational training, primary education, or no education), information on maternal education was missing for 83 participants (3.0%). Mothers included in the follow‐up studies were older, more frequently of Dutch origin, and higher educated compared to mothers who only participating during the fetal period (Kooijman et al., 2016).

**TABLE 1:** Overview of the fearful temperament, anxiety, and peer interaction variables and the covariates in the current sample (*n* = 2730).

	*M*	*SD*	Min	Max	Skewness	Missing (%)
Parent‐reported fearful temperament score (IBQ‐R) at 6 months	0.36	0.29	0	1.87	1.33	0
Age at 6‐month fearful temperament assessment (in months)	6.64	1.02	4.52	11.91	2.08	13.08
Observed fearful temperament score (Lab‐TAB) at 3 years	−0.03	0.36	−1.16	1.43	0.11	83.85
Age at 3‐year fearful temperament assessment (in months)	37.11	1.2	34.74	43.19	1.36	82.86
Parent‐reported anxiety score (CBCL) at 13 years	1.12	1.59	0	11	2.02	0
Age at parent‐reported anxiety assessment (in years)	13.49	0.32	12.54	16.22	2.41	0
Self‐reported anxiety score (YSR) at 13 years	2.09	1.88	0	10	1.07	6.04
Age at self‐reported anxiety assessment (in years)	13.54	0.35	12.57	16.32	2.43	4.95
Peer interaction variables						
Victimization score at 7 years	5.27	2.24	1	20	2.41	16.15
Age at victimization assessment (in years)	8.1	0.16	7.51	9.99	4.28	15.46
Friendship quality score at 9 years	3.53	0.63	1	4.78	−0.46	16.96
Age at friendship quality assessment (in years)	9.76	0.28	8.72	12.4	2.04	12.56
Negative feelings score during cyberball task at 9 years	2.86	0.82	1	5	0.12	18.46
Negative feelings score after cyberball task at 9 years	1.55	0.43	1	4.14	1.14	18.46
Angry facial expressions score during cyberball task at 9 years	1.24	0.2	0	3.1	2.56	18.46
Sad facial expressions score during cyberball task at 9 years	1.56	0.16	0	2.68	1.97	18.46
Age at cyberball assessment (in years)	9.73	0.25	8.55	11.84	1.61	18.46
Covariates						
Age mother at birth child (in years)	32.28	4.37	16.85	46.85	−0.29	0
Puberty score at 13 years	2.37	0.75	1	4	−0.11	16.89

Abbreviations: CBCL, Child Behavior Checklist; IBQ‐R, Infant Behavior Questionnaire‐Revised; Lab‐TAB, Laboratory Temperament Assessment Battery; YSR, Youth Self‐Report.

### Measures

#### Fearful temperament

##### Parent‐reported fearful temperament

Fearful temperament was measured with an adapted version of the Infant Behavior Questionnaire‐Revised (IBQ‐R) (Gartstein & Rothbart, 2003), which was filled out by the mother when children were 6 months old. The adapted version (171 items) (Roza et al., 2008) included six subscales, of which we only used the Fear subscale (15 items) measuring fear of new objects and unfamiliar persons (e.g. “When your child was in touch with an unknown adult, how often did your child refuse to go to the unknown person?”). The items were scored on a 3‐point Likert scale (0 = never present, 1 = sometimes present, 2 = often present) in this adapted version. Internal consistencies for the Fear subscale in the full sample (*n* = 3751) was 0.86, which is comparable to the internal consistency of the original IBQ‐R (Gartstein & Rothbart, 2003; Roza et al., 2008).

##### Observed fearful temperament

Early‐life fearful temperament was also measured with behavioral observations from the Laboratory Temperament Assessment Battery (Lab‐TAB) (Gagne et al., 2011) when children were 3 years old in a subset of the sample that visited the lab (*n* = 862). Fear (e.g. intensity of fear expressions, distress vocalizations, withdrawal, gaze aversion) was observed in the “jumping spider” episode in response to physical contact with a hidden jumping spider toy and in the “stranger approach” episode which is a social interaction with an unfamiliar adult wearing a hat and sunglasses (Gagne et al., 2011), for a complete overview of the Generation R procedure see Zwirs et al. (2015). Coders (*n* = 25) were blind to all other measures, were extensively trained, and reliability (interobserver reliability ranged from 0.66 to 0.97) was established before data were coded (Kok et al., 2013). The behavioral scores were standardized and averaged across the two episodes. The internal consistency was 0.80 for the “jumping spider” episode and 0.62 for the “stranger approach” episodes, the subscales did not correlate, *r* = 0.03, *p* = 0.50.

#### Anxiety

Anxiety symptoms were measured with the DSM anxiety scale of the Child Behavior Checklist (CBCL) (Achenbach & Rescorla, 2001), which was filled out by the primary caregiver when children were 13 years old. The full CBCL consists of 112 items, the DSM anxiety scale consists of 6 items measuring anxiety symptoms such as “Too fearful or anxious” (Achenbach et al., 2003; Ebesutani et al., 2010). The anxiety scale does not measure one specific type of anxiety (e.g. social anxiety or generalized anxiety) but assesses multiple types, therefore we refer to this as “general anxiety”. The items were scored on a 3‐point Likert scale (1 = not true, 2 = somewhat or sometimes true, 3 = very true or often true). The internal consistency for the DSM anxiety subscale in the full sample (*n* = 4670) was 0.70.

#### Peer interaction variables

##### Victimization

Victimization was measured with a questionnaire on bullying and victimization (Alsaker & Valkanover, 2000), which was filled out by the primary caregiver when children were around 7 years old. The rationale for assessing victimization this early is that bullying is already common at the start of elementary school and is related to the development of psychosocial problems in young children (Arseneault et al., 2006; Kochenderfer & Ladd, 1997; Perren & Alsaker, 2006). The victimization subscale consists of four items related to general, verbal, physical, and relational victimization. Items were scored on a five‐point Likert scale (1 = never, 2 = seldom: only once or twice, 3 = 2 or 3 times a month, 4 = about once a week, 5 = several times a week) and summed. The internal consistency for the victimization subscale in the full sample (*n* = 4715) was 0.82.

##### Friendship quality

Friendship quality was measured with an adapted version of the Friendship Quality Questionnaire (Parker & Asher, 1993), which was filled out by the children when they were 9 years. The adapted version consists of 10 items (e.g. “We give each other compliments”) that were scored on a 3‐point Likert scale (1 = not true, 2 = somewhat true, 3 = very true) and averaged. The internal consistency of this measure in the full sample (*n* = 4372) was 0.67.

##### Social exclusion

Responses to social exclusion were measured with the Cyberball task (Williams et al., 2000) when children were 9 years. Children were led to believe that they would play an online ball‐tossing game with two unfamiliar same‐sex peers on a desktop during the lab visit. Children were included in the first six ball tosses of the task (“fair period”) and then excluded in the remaining 36 ball tosses (“unfair period”). Afterwards, they filled out a questionnaire about their mood, self‐esteem, feelings of control and belonging during the task, and about their mood, self‐esteem, and meaningful existence after the task. These items formed two scales as confirmed by factor analysis: feelings during (6 items) and after (7 items) the task (Mulder et al., 2023). The internal consistency of these scales in the full sample (*n* = 4813) were respectively 0.81 and 0.72, and these scales were correlated, *r* = 0.25, *p* < 0.001.

Facial expressions were recorded with a webcam throughout the task and coded by four raters for anger, contempt, and sadness (Mulder et al., 2023). Coding was adapted from Ekman's coding of discrete muscle movements (Ekman & Friesen, 1982). Angry and sad expressions were continuously scored and an area under the curve (AUC) represented the duration and intensity of the emotion during inclusion and exclusion separately. The AUCs during the unfair period were corrected for the AUC during the fair period and then the values were logarithmically scaled (Mulder et al., 2023).

#### Other variables

Sex was obtained from birth records and maternal age and education from questionnaires. Maternal education categorized into two categories: university (university of applied sciences, university) and other education (secondary education, vocational training, primary education, or no education). Child national origin was based on the birth country of the parents and categorized as Dutch (both parents were born in the Netherlands) or not Dutch. Child age at the anxiety symptoms measurement was calculated using the birth date. Puberty was assessed with the Pubertal Development Scale (Carskadon & Acebo, 1993) at age 13 (internal consistency was 0.86 for boys [*n* = 4937] and 0.81 for girls [*n* = 4808]).

### Analysis

Robust linear regression models were used in R to account for non‐normally distributed anxiety symptoms, using the MASS package (Ripley et al., 2002). All independent variables were standardized. First, we tested whether fearful temperament and its interaction with sex were related to adolescent anxiety with a linear regression model with anxiety as dependent variable, and fearful temperament and sex as independent variables in step 1. We added the interaction between fearful temperament and sex in step 2 and the covariates (age and pubertal status at the anxiety symptoms measurement, child national origin, maternal age, and maternal education) in step 3. We ran these models separately for parent‐reported and observed fearful temperament (*r* = 0.06, *p* = 0.18), and continued the analyses with the variables that showed a significant association with anxiety (only parent‐reported fearful temperament).

Second, we tested in separate analyses whether the peer interaction variables moderated the association between fearful temperament and anxiety. We ran linear regression models with anxiety as dependent variable, fearful temperament, one of the peer interaction variables, and sex as independent variables in step 1. We added the interaction between fearful temperament and the peer interaction variable(s) in step 2 and the covariates (age and pubertal status at the anxiety symptoms measurement, child national origin, maternal age, and maternal education) in step 3. We ran these models separately for victimization, friendship quality, negative feelings (during & after social exclusion together), and negative facial expressions during social exclusion (sad & angry together), resulting in 4 sets of analyses. Since the interaction between fearful temperament and sex was not significant, we included sex only as a main effect.

Participants were only included when they had data on fearful temperament and anxiety. If more than 25% of the items were missing for parent‐reported fearful temperament and anxiety, data for that participant were excluded. For observed fearful temperament, more than 50% of the data had to be present. If fewer items were missing than these thresholds, weighted scores were calculated. For the other variables, scores were calculated regardless of missing items and adjusted for the number of non‐missing items. Missing data on the other variables were imputed using the mice package (van Buuren & Groothuis‐Oudshoorn, 2011) with a maximum of 100 iterations creating 30 datasets. Results were based on pooled estimates of the multiple imputed sets. Inverse probability weighting was used in all analyses to account for selection bias (Seaman & White, 2013) with sex, child national origin, maternal age, and maternal education predicting inclusion in the final sample (*n* = 2730). Post‐hoc linear regression models were run separately for children with high versus low fearful temperament scores (median split), in case of a significant interaction between fearful temperament and a peer interaction variable.

Finally, we ran a series of pre‐registered (sensitivity) analyses that are described in the Supporting Information: repeating the main analysis with i) peer‐reported victimization and friendships (Appendix S4), ii) parent‐reported fearful temperament and self‐reported anxiety (Appendix S5), iii) all peer interaction variables in one model (Appendix S6), iv) without outliers (values that are 3 SDs above/below the mean; Appendix S7), and v) depressive symptoms as additional covariate (Appendix S8).

## RESULTS

### Fearful temperament‐anxiety association

Children with higher scores on parent‐reported fearful temperament at 6 months showed more anxiety symptoms when they were 13 years, *β* = 0.07, *p* < 0.001. This association was not moderated by sex, *β* = −0.07, *p* = 0.07, and remained the same when the covariates were added (Table 2). Children with higher scores on observed fearful temperament at 3 years did not show more anxiety symptoms when they were 13 years, *β* = 0.01, *p* = 0.77 (Appendix S3).

**TABLE 2:** Results of the robust linear regression model with parent‐reported fearful temperament as independent variable and anxiety as dependent variable.

	*beta*	*SE*	*t*	*df*	*p*
Step 1					
Intercept	0.77	0.03	26.13	2703.57	<0.001
Fearful temperament	0.07	0.02	3.46	2707.48	<0.001
Sex: Girl versus boy	0.10	0.04	2.30	2715.99	0.02
Step 2					
Intercept	0.78	0.03	26.26	2696.92	<0.001
Fearful temperament	0.11	0.03	3.68	2709.27	<0.001
Sex: Girl versus boy	0.10	0.04	2.34	2715.17	0.02
Fearful temperament × Sex	−0.07	0.04	−1.85	2706.75	0.07
Step 3					
Intercept	0.85	0.08	10.01	771.15	<0.001
Fearful temperament	0.13	0.03	4.22	2701.15	<0.001
Sex: Girl versus boy	0.10	0.05	1.84	1617.38	0.07
Temperament × Sex	−0.08	0.04	−1.95	2702.94	0.05
Age (in years) at CBCL	0.03	0.02	1.27	2681.82	0.21
Puberty status at CBCL	−0.001	0.04	−0.03	528.55	0.98
Maternal age	0.07	0.02	3.48	2681.30	<0.001
Maternal education: Other versus university	−0.03	0.05	−0.69	2172.09	0.49
Child national origin: Non‐Dutch versus Dutch	−0.04	0.05	−0.92	2633.21	0.36

Abbreviation: CBCL, Child Behavior Checklist.

### Sensitivity analyses

Sensitivity analyses for this research question showed that the parent‐reported fearful temperament‐anxiety association remained significant when self‐reported anxiety symptoms were used, *β* = 0.10, *p* < 0.001 (Appendix S5), and when depressive symptoms at age 13 were added as an additional covariate, *β* = 0.08, *p* = 0.001 (Appendix S8).

### Peer interaction variables as moderator

**FIGURE 2:**
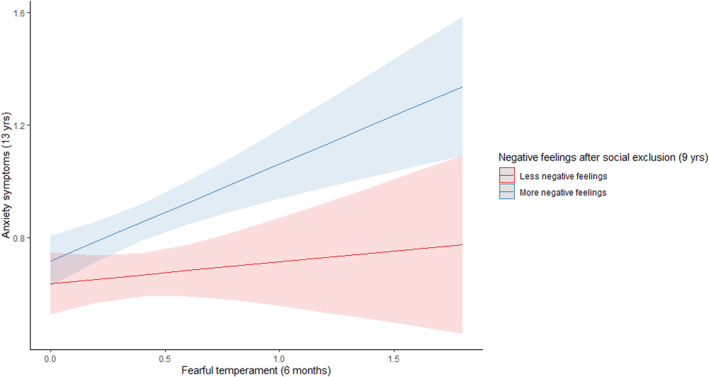
Negative feelings after social exclusion (measured at 9 years) moderated the association between parent‐reported fearful temperament (measured at 6 months) and anxiety symptoms (measured at 13 years).

Children who experienced more victimization, lower friendship quality, more negative feelings after social exclusion, and fewer sad facial expressions during social exclusion reported more anxiety symptoms, βs>|0.05|, *p*s < 0.02 (Appendix S2). Victimization, friendship quality, negative feelings during social exclusion, sad and angry facial expressions did not moderate the association between parent‐reported fearful temperament and anxiety symptoms, βs<|0.03|, *p*s > 0.27 (Appendix S2). However, negative feelings after social exclusion moderated the association between parent‐reported fearful temperament at 6 months and anxiety symptoms at 13 years, *β* = 0.05, *p* = 0.04 (Table 3). The association between parent‐reported fearful temperament and anxiety symptoms was stronger for children with more negative feelings after social exclusion, *β* = 0.18, *p* = 0.005, than for children with less negative feelings after social exclusion, *β* = 0.07, *p* = 0.28 (Figure 2).

**TABLE 3:** Results of the robust linear regression model with fearful temperament as independent variable, anxiety as dependent variable and negative feelings as moderator.

	*beta*	*SE*	*t*	df	*p*
Step 1					
Intercept	0.79	0.03	26.38	2667.92	<0.001
Fearful temperament	0.07	0.02	3.58	2646.70	<0.001
Negative feelings during cyberball	0.01	0.02	0.31	400.01	0.76
Negative feelings after cyberball	0.14	0.02	5.84	317.07	<0.001
Sex: Girl versus boy	0.09	0.04	2.15	2678.29	0.03
Step 2					
Intercept	0.79	0.03	26.48	2671.81	<0.001
Fearful temperament	0.08	0.02	4.03	2141.85	<0.001
Negative feelings during cyberball	0.01	0.02	0.32	443.11	0.75
Negative feelings after cyberball	0.14	0.02	5.76	367.89	<0.001
Sex: Girl versus boy	0.09	0.04	2.12	2642.27	0.03
Fearful temperament × Negative feelings during cyberball	0.02	0.02	0.83	286.99	0.41
Fearful temperament × Negative feelings after cyberball	0.05	0.02	2.10	176.72	0.04
Step 3					
Intercept	0.85	0.09	9.78	560.31	<0.001
Fearful temperament	0.10	0.02	4.68	2203.38	<0.001
Negative feelings during cyberball	0.001	0.02	0.03	501.46	0.97
Negative feelings after cyberball	0.14	0.02	5.79	390.59	<0.001
Sex: Girl versus boy	0.08	0.05	1.59	1559.01	0.11
Age at CBCL	0.03	0.02	1.66	2648.58	0.10
Puberty status at CBCL	−0.002	0.04	−0.05	416.19	0.96
Child national origin: Non‐Dutch versus Dutch	−0.04	0.05	−0.91	2575.47	0.36
Maternal age	0.07	0.02	3.50	2589.52	<0.001
Maternal education: Other versus university	−0.02	0.05	−0.47	1943.98	0.64
Fearful temperament × Negative feelings during cyberball	0.02	0.02	0.93	318.43	0.35
Fearful temperament × Negative feelings after cyberball	0.05	0.02	2.18	177.72	0.03

Abbreviation: CBCL, Child Behavior Checklist.

#### Sensitivity analyses

We checked if the interaction between parent‐reported fearful temperament and negative feelings after social exclusion was robust against changes in the analysis. This interaction remained similar when all peer interaction variables were included in one model, *β* = 0.05, *p* = 0.03 (Appendix S6). The interaction did not remain significant with observed instead of parent‐reported fearful temperament, *β* = −0.003, *p* = 0.96 (Appendix S3), when self‐reported anxiety symptoms were used instead of parent‐reported anxiety symptoms, *β* = 0.06, *p* = 0.12 (Appendix S5), when outliers were excluded and imputed (range: 0%–2.09% outliers per variable), *β* = 0.02, *p* = 0.35 (Appendix S7), and when depression was included as additional covariate, *β* = 0.01, *p* = 0.55 (Appendix S8).

  For observed fearful temperament, only the interaction with friendship quality was significant, *β* = 0.11, *p* = 0.04 (Appendix S3).

## DISCUSSION

The goal of this study was to replicate the fearful temperament‐anxiety association in a large cohort study, explore sex as a moderator of this association, and to investigate four distinct peer interaction variables as moderators of the fearful temperament‐anxiety association. For this, we used data from a prospective cohort study, the Generation R Study. Findings indicated that children with higher parent‐reported, but not observed, fearful temperament showed more anxiety symptoms as adolescents. Moreover, this association was stronger in adolescents who reported more negative feelings after social exclusion. More victimization, lower friendship quality, and sad facial expressions during social exclusion were also related to increased anxiety symptoms, but did not moderate the fearful temperament‐anxiety association.

We replicated the well‐established finding that early‐life highly fearful temperament is related to later anxiety (Clauss & Blackford, 2012; Garcia‐Lopez et al., 2020; Hudson et al., 2019; Rapee, 2014; Sandstrom et al., 2020) for parent‐reported but not for observed fearful temperament. Even though most studies have found the strongest relation in patient samples with social anxiety disorder (Clauss & Blackford, 2012; Garcia‐Lopez et al., 2020; Rapee, 2014), here we demonstrated the same association with parent‐reported fearful temperament and general anxiety symptoms in a community sample. We also showed that the fearful temperament‐anxiety association was not moderated by sex, in line with previous studies (Hudson et al., 2019; Sandstrom et al., 2020).

Here we showed that negative feelings after social exclusion moderated the parent‐reported fearful temperament‐anxiety association. Children who exhibited both higher parent‐reported fearful temperament at 6 months and negative feelings after social exclusion at 9 years showed more anxiety symptoms at 13 years. Social exclusion is a hurtful experience that challenges our sense of belonging and social connectedness (Hartgerink et al., 2015; Williams et al., 2000), especially in adolescence (Gunther Moor et al., 2010) when peer interactions become increasingly important (Andrews et al., 2021; Blakemore, 2018; Blakemore & Mills, 2014). Negative responses to social exclusion are associated with the development and maintenance of psychopathology (Reinhard et al., 2020). We found the interaction for negative feelings *after* and not *during* social exclusion, suggesting that responses after the situation might have more effect than responses during. In line with this, we also found that negative facial expressions *during* the task did not moderate the fearful temperament‐anxiety association. In contrast, children with a higher risk for anxiety symptoms may have had more prolonged responses. This could be related to rumination, which is a transdiagnostic risk factor for anxiety in adolescents (Kraft et al., 2023; Schafer et al., 2017), as opposed to acute stress. Potentially, children with a highly fearful temperament may benefit from learning how to cope with social rejection. Future research could test whether focusing on coping with social exclusion could maybe improve effectiveness of interventions. It should be noted that the interaction did not remain significant after outliers were removed. The cut‐off of 3 SDs might have been too strict, because the values were not invalid and not very different from the other values in the tail of the distribution. As our design precludes conclusions on causal associations and the association was not significant in all sensitivity analyses, future studies are needed to support our findings.

More victimization, lower friendship quality, and fewer sad facial expressions during social exclusion were related to adolescent anxiety symptoms. The finding that *fewer* sad facial expressions were related to anxiety is line with the finding that negative facial expressions were related to internalizing symptoms in an overlapping sample, which was interpreted as a possible effect of emotion suppression (Mulder et al., 2023). Victimization, friendship quality, and facial expressions did not moderate the fearful temperament‐anxiety association. This suggests that these peer interaction variables did not exacerbate or temper the association. Previously, victimization did show an interaction with fearful temperament in predicting social anxiety (Degnan et al., 2010) but not in predicting internalizing symptoms (Boyd et al., 2003). So, it could be that victimization is more related to social anxiety and not to general anxiety as measured in the current study. Regarding friendships, it is possible that not so much the quality of friendships is relevant here, but more that the friends' characteristics play a role: some friends might weaken the fearful temperament‐anxiety association by teaching social skills, whereas other friends have a highly fearful temperament themselves as well and might not provide an example of alternative behavior (Degnan et al., 2010).

These associations were only present for parent‐reported fearful temperament as measured with the IBQ‐R (Gartstein & Rothbart, 2003), not for observed fearful temperament as measured with the Lab‐TAB (Gagne et al., 2011) in a smaller sample. Meta‐analytic evidence suggests that both parent‐reported and observed fearful temperament are related to anxiety (Clauss & Blackford, 2012; Sandstrom et al., 2020), but the use of questionnaire data has also been critiqued (Runze & Van IJzendoorn, 2023). As observational measures of fearful temperament, the “stranger approach” and “jumping spider” episodes of the Lab‐TAB might not be ideal, because many children respond to these high‐threat situations (Lissek et al., 2006). However, some children also show fear in low‐threat situations, and these episodes (e.g. “clowns” or “puppets” from the Lab‐TAB) might be more useful to provoke individual differences in fearful temperament (Buss & McDoniel, 2016). In line with this, other studies that did find an association between observed fearful temperament and anxiety have used different situations to observe fearful temperament, such as a toy robot and a tunnel (Fox et al., 2023), which are also less threatening.

This prospective cohort study included data on fearful temperament, different forms of peer interactions, and anxiety in a large community sample spanning 13 years. Some limitations should be considered. First, it should be noted that children were asked after the task to rate their feelings during the task (retrospective report) and at that moment (concurrent report). Nevertheless, factor analysis showed that the feelings during and after the task were best explained by two separate factors (Mulder et al., 2023). Second, the interaction between fearful temperament and negative feelings after social exclusion was not significant in most sensitivity analyses. This could be due to the issues with the Lab‐TAB episodes as described above, due to the definition of outliers, because of the smaller sizes or because of the high comorbidity between anxiety and depressive symptoms. Third, only parent‐report victimization was available for the full sample, instead of self‐report or peer nominations, and parents might not always be aware of victimization.

## CONCLUSION

To conclude, this large prospective cohort study showed that high early‐life parent‐reported fearful temperament is related to adolescent anxiety symptoms, and that this association is not moderated by sex. Moreover, this parent‐reported fearful temperament‐anxiety association was stronger in adolescents who reported more negative feelings after social exclusion. We discussed potential avenues for future research, such as examining possibly amplifying treatment with a module on coping with social exclusion.

## AUTHOR CONTRIBUTIONS

**Anita Harrewijn**: Conceptualization; formal analysis; funding acquisition; investigation; methodology; visualization; writing – original draft. **Rosa H. Mulder**: Methodology; writing – review & editing. **Marinus H. van IJzendoorn**: Methodology; writing – review & editing. **Matthias J. Wieser**: Investigation; writing – review & editing. **Pauline W. Jansen**: Conceptualization; methodology; resources; supervision; writing – review & editing.

## CONFLICT OF INTEREST STATEMENT

The authors have no conflict of interest.

## ETHICAL CONSIDERATIONS

This study was performed in line with the principles of the Declaration of Helsinki. Study procedures were approved by the Medical Ethics Committee of the Erasmus University Medical Center. Parents provided informed consent for their children, and children aged 12 years and older also signed their own consent form, in accordance with Dutch law.

## Supporting information

Supporting Information S1

## Data Availability

The data that support the findings of this study are available from Generation R. Restrictions apply to the availability of these data, which were used under license for this study.
