# Supplementary material for: Four distinct peer interaction variables as moderators of the fearful temperament‐anxiety association, using data from the Generation R Study
Source: JCPP Adv. 2024 Jun 19;5(1):e12254. doi: 10.1002/jcv2.12254 (PMC11889650; doi:10.1002/jcv2.12254)
Supplement: Supplementary file 1 — Supporting Information S1 [file JCV2-5-e12254-s001.docx]

**Supporting Information**

**Four distinct peer interaction variables as moderators of the fearful temperament-anxiety association, using data from the Generation R Study**

**Overview Supporting Information**

| Analysis in | RQ 1 (temp-anx) | RQ2 (moderator) | Independent variable | Moderator | Dependent variable | Analysis note |
| --- | --- | --- | --- | --- | --- | --- |
| Main text 1 | x | x | Parent-reported temperament | Parent-reported victimization Self-reported friendship quality Self-reported negative feelings Observed negative facial expressions | Parent-reported anxiety |  |
| Main text 2 | x |  | Observed temperament |  | Parent-reported anxiety |  |
| Appendix S1 | Correlation table | |  |  |  |  |
| Appendix S2 |  | x | Parent-reported temperament | Parent-reported victimization Self-reported friendship quality Self-reported negative feelings Observed negative facial expressions | Parent-reported anxiety |  |
| Appendix S3 | x | x | Observed temperament | Parent-reported victimization Self-reported friendship quality Self-reported negative feelings Observed negative facial expressions | Parent-reported anxiety |  |
| Appendix S4 |  | x | Parent-reported temperament | Peer-reported victimization  Peer-reported friendships | Parent-reported anxiety |  |
| Appendix S5 | x | x | Parent-reported temperament |  | Self-reported anxiety |  |
| Appendix S6 |  | x | Parent-reported temperament | Parent-reported victimization Self-reported friendship quality Self-reported negative feelings Observed negative facial expressions | Parent-reported anxiety | All in one model |
| Appendix S7 | x | x | Parent-reported temperament | Parent-reported victimization Self-reported friendship quality Self-reported negative feelings Observed negative facial expressions | Parent-reported anxiety | Without outliers |
| Appendix S8 | x | x | Parent-reported temperament | Parent-reported victimization Self-reported friendship quality Self-reported negative feelings Observed negative facial expressions | Parent-reported anxiety | Depressive symptoms as additional covariate |

Note: The findings of the variables in black are shown in tables and in grey are only mentioned in the text. RQ = research question, temp = temperament, anx = anxiety.

**Appendix S1. Correlation table with all variables included in the study**

Table S1. Correlation table with all variables included in the study.

|  |  | Observed temperament | Parent-reported anxiety | Age at parent-reported anxiety assessment | Self-reported anxiety | Age at self-reported anxiety assessment | Victimization | Friendship quality | Negative feelings during Cyberball task | Negative feelings after Cyberball task | Angry facial expressions during Cyberball task | Sad facial expressions during Cyberball task | Age mother at birth child | Puberty at 13 years |
| --- | --- | --- | --- | --- | --- | --- | --- | --- | --- | --- | --- | --- | --- | --- |
| Parent-reported temperament | *r* | 0.06 | 0.08 | 0.04 | 0.08 | 0.03 | 0 | -0.01 | -0.05 | 0 | 0.02 | 0 | -0.15 | 0.13 |
|  | *p* | 0.18 | <0.001 | 0.04 | <0.001 | 0.08 | 0.88 | 0.71 | 0.01 | 0.82 | 0.34 | 0.95 | <0.001 | <0.001 |
|  | *n* | 532 | 2730 | 2851 | 2619 | 2723 | 2846 | 2655 | 2740 | 2740 | 2740 | 2740 | 4182 | 2316 |
| Observed temperament | *r* |  | -0.02 | 0.05 | -0.01 | 0.05 | -0.06 | -0.04 | -0.03 | 0.01 | -0.03 | -0.05 | -0.03 | 0.02 |
|  | *p* |  | 0.68 | 0.23 | 0.87 | 0.23 | 0.15 | 0.26 | 0.46 | 0.88 | 0.39 | 0.19 | 0.48 | 0.65 |
|  | *n* |  | 643 | 659 | 622 | 639 | 662 | 639 | 624 | 624 | 624 | 624 | 801 | 561 |
| Parent-reported anxiety | *r* |  |  | 0.02 | 0.44 | 0.02 | 0.15 | -0.04 | 0.02 | 0.12 | 0 | -0.05 | 0.03 | 0.04 |
|  | *p* |  |  | 0.23 | <0.001 | 0.14 | <0.001 | 0.03 | 0.19 | <0.001 | 0.93 | 0.002 | 0.06 | 0.01 |
|  | *n* |  |  | 4717 | 4407 | 4466 | 3631 | 3601 | 3550 | 3550 | 3550 | 3550 | 4716 | 3888 |
| Age at parent-reported anxiety assessment | *r* |  |  |  | 0.03 | 0.92 | 0 | 0 | -0.01 | 0.01 | 0 | -0.04 | -0.05 | 0.18 |
|  | *p* |  |  |  | 0.07 | <0.001 | 0.84 | 0.82 | 0.75 | 0.37 | 0.87 | 0.01 | <0.001 | <0.001 |
|  | *n* |  |  |  | 4476 | 4681 | 3781 | 3723 | 3679 | 3679 | 3679 | 3679 | 4961 | 3948 |
| Self-reported anxiety | *r* |  |  |  |  | 0.03 | 0.1 | 0 | 0.08 | 0.16 | 0 | -0.03 | -0.02 | 0.12 |
|  | *p* |  |  |  |  | 0.03 | <0.001 | 0.95 | <0.001 | <0.001 | 0.90 | 0.10 | 0.19 | <0.001 |
|  | *n* |  |  |  |  | 4520 | 3524 | 3512 | 3414 | 3414 | 3414 | 3414 | 4519 | 3961 |
| Age at self-reported anxiety assessment | *r* |  |  |  |  |  | -0.01 | 0.01 | 0 | 0 | 0 | -0.05 | -0.07 | 0.19 |
|  | *p* |  |  |  |  |  | 0.64 | 0.54 | 0.79 | 0.81 | 0.98 | 0.004 | <0.001 | <0.001 |
|  | *n* |  |  |  |  |  | 3651 | 3605 | 3512 | 3512 | 3512 | 3512 | 4728 | 3983 |
| Victimization | *r* |  |  |  |  |  |  | -0.07 | 0.04 | 0.08 | 0.01 | -0.02 | -0.02 | 0.01 |
|  | *p* |  |  |  |  |  |  | <0.001 | 0.04 | <0.001 | 0.54 | 0.37 | 0.23 | 0.52 |
|  | *n* |  |  |  |  |  |  | 3513 | 3344 | 3344 | 3344 | 3344 | 4714 | 3132 |
| Friendship quality | *r* |  |  |  |  |  |  |  | -0.09 | -0.17 | 0.04 | -0.02 | -0.01 | 0.13 |
|  | *p* |  |  |  |  |  |  |  | <0.001 | <0.001 | 0.03 | 0.32 | 0.38 | <0.001 |
|  | *n* |  |  |  |  |  |  |  | 3641 | 3641 | 3641 | 3641 | 4372 | 3126 |
| Negative feelings during Cyberball task | *r* |  |  |  |  |  |  |  |  | 0.26 | 0.06 | 0.09 | 0.04 | -0.02 |
|  | *p* |  |  |  |  |  |  |  |  | <0.001 | <0.001 | <0.001 | 0.005 | 0.22 |
|  | *n* |  |  |  |  |  |  |  |  | 4813 | 4813 | 4813 | 4813 | 2997 |
| Negative feelings after Cyberball task | *r* |  |  |  |  |  |  |  |  |  | 0 | 0 | -0.02 | 0.02 |
|  | *p* |  |  |  |  |  |  |  |  |  | 0.86 | 0.96 | 0.16 | 0.18 |
|  | *n* |  |  |  |  |  |  |  |  |  | 4813 | 4813 | 4813 | 2997 |
| Angry facial expressions during Cyberball task | *r* |  |  |  |  |  |  |  |  |  |  | 0.13 | -0.02 | 0.03 |
|  | *p* |  |  |  |  |  |  |  |  |  |  | <0.001 | 0.11 | 0.13 |
|  | *n* |  |  |  |  |  |  |  |  |  |  | 4813 | 4813 | 2997 |
| Sad facial expressions during Cyberball task | *r* |  |  |  |  |  |  |  |  |  |  |  | 0.02 | -0.03 |
|  | *p* |  |  |  |  |  |  |  |  |  |  |  | 0.19 | 0.11 |
|  | *n* |  |  |  |  |  |  |  |  |  |  |  | 4813 | 2997 |
| Age mother at birth child | *r* |  |  |  |  |  |  |  |  |  |  |  |  | -0.06 |
|  | *p* |  |  |  |  |  |  |  |  |  |  |  |  | <0.001 |
|  | *n* |  |  |  |  |  |  |  |  |  |  |  |  | 3982 |

**Appendix S2. Main analysis with victimization, friendship quality, and facial expressions as moderators**

Table S2a. Results of the robust linear regression model with anxiety as dependent variable and victimization as moderator.

|  | *beta* | *SE* | *t* | df | *p* |
| --- | --- | --- | --- | --- | --- |
| *Step 1* |  |  |  |  |  |
| Intercept | 0.78 | 0.03 | 26.36 | 2626.78 | <0.001 |
| Temperament | 0.07 | 0.02 | 3.63 | 2643.90 | <0.001 |
| Victimization | 0.16 | 0.03 | 5.49 | 96.81 | <0.001 |
| Sex: girl vs boy | 0.10 | 0.04 | 2.33 | 2686.65 | 0.02 |
| *Step 2* |  |  |  |  |  |
| Intercept | 0.78 | 0.03 | 26.33 | 2615.62 | <0.001 |
| Temperament | 0.08 | 0.02 | 3.78 | 1518.75 | <0.001 |
| Victimization | 0.16 | 0.03 | 5.51 | 109.60 | <0.001 |
| Sex: girl vs boy | 0.10 | 0.04 | 2.34 | 2675.14 | 0.02 |
| Temperament*Victimization | 0.03 | 0.03 | 1.11 | 72.53 | 0.27 |
| *Step 3* |  |  |  |  |  |
| Intercept | 0.85 | 0.08 | 10.13 | 764.83 | <0.001 |
| Temperament | 0.09 | 0.02 | 4.42 | 1833.30 | <0.001 |
| Victimization | 0.16 | 0.03 | 5.52 | 118.53 | <0.001 |
| Sex: girl vs boy | 0.10 | 0.05 | 1.88 | 1505.58 | 0.06 |
| Age at CBCL | 0.03 | 0.02 | 1.52 | 2625.55 | 0.13 |
| Puberty status at CBCL | -0.01 | 0.04 | -0.14 | 494.48 | 0.89 |
| Child national origin: non-Dutch vs Dutch | -0.02 | 0.04 | -0.50 | 2499.70 | 0.61 |
| Maternal age | 0.07 | 0.02 | 3.39 | 2495.20 | <0.001 |
| Maternal education: other vs university | -0.05 | 0.05 | -1.00 | 2125.23 | 0.32 |
| Temperament*Victimization | 0.03 | 0.03 | 1.16 | 75.69 | 0.25 |

Note: CBCL = Child Behavior Checklist.

Table S2b. Results of the robust linear regression model with anxiety as dependent variable and friendship quality as moderator.

|  | *beta* | *SE* | *t* | df | *p* |
| --- | --- | --- | --- | --- | --- |
| *Step 1* |  |  |  |  |  |
| Intercept | 0.76 | 0.03 | 25.11 | 2679.24 | <0.001 |
| Temperament | 0.07 | 0.02 | 3.35 | 2706.99 | <0.001 |
| Friendship quality | -0.06 | 0.02 | -2.53 | 428.33 | 0.01 |
| Sex: girl vs boy | 0.13 | 0.04 | 2.92 | 2545.86 | 0.004 |
| *Step 2* |  |  |  |  |  |
| Intercept | 0.76 | 0.03 | 25.05 | 2664.43 | <0.001 |
| Temperament | 0.07 | 0.02 | 3.37 | 2689.98 | 0.001 |
| Friendship quality | -0.06 | 0.02 | -2.50 | 476.25 | 0.01 |
| Sex: girl vs boy | 0.13 | 0.04 | 2.92 | 2525.73 | 0.003 |
| Temperament*Friendship quality | -0.01 | 0.02 | -0.44 | 216.61 | 0.66 |
| *Step 3* |  |  |  |  |  |
| Intercept | 0.83 | 0.09 | 9.77 | 754.90 | <0.001 |
| Temperament | 0.08 | 0.02 | 4.01 | 2682.58 | <0.001 |
| Friendship quality | -0.06 | 0.02 | -2.39 | 529.62 | 0.02 |
| Sex: girl vs boy | 0.12 | 0.06 | 2.27 | 1380.03 | 0.02 |
| Age at CBCL | 0.03 | 0.02 | 1.36 | 2664.60 | 0.17 |
| Puberty status at CBCL | -0.001 | 0.04 | -0.02 | 517.12 | 0.98 |
| Child national origin: non-Dutch vs Dutch | -0.05 | 0.05 | -1.08 | 2628.78 | 0.28 |
| Maternal age | 0.07 | 0.02 | 3.26 | 2660.89 | 0.001 |
| Maternal education: other vs university | -0.03 | 0.05 | -0.72 | 2188.40 | 0.47 |
| Temperament*Friendship quality | -0.01 | 0.02 | -0.52 | 233.28 | 0.61 |

Note: CBCL = Child Behavior Checklist.

Table S2c. Results of the robust linear regression model with anxiety as dependent variable and negative facial expressions as moderators.

|  | *beta* | *SE* | *t* | df | *p* |
| --- | --- | --- | --- | --- | --- |
| *Step 1* |  |  |  |  |  |
| Intercept | 0.78 | 0.03 | 26.19 | 2677.08 | <0.001 |
| Temperament | 0.07 | 0.02 | 3.52 | 2702.02 | <0.001 |
| Angry facial expressions | -0.003 | 0.02 | -0.11 | 380.90 | 0.91 |
| Sad facial expressions | -0.05 | 0.02 | -2.31 | 616.44 | 0.02 |
| Sex: girl vs boy | 0.09 | 0.04 | 2.16 | 2704.16 | 0.03 |
| *Step 2* |  |  |  |  |  |
| Intercept | 0.78 | 0.03 | 26.19 | 2683.93 | <0.001 |
| Temperament | 0.07 | 0.02 | 3.60 | 2680.84 | <0.001 |
| Angry facial expressions | -0.003 | 0.02 | -0.13 | 370.67 | 0.90 |
| Sad facial expressions | -0.05 | 0.02 | -2.18 | 751.48 | 0.03 |
| Sex: girl vs boy | 0.09 | 0.04 | 2.18 | 2698.21 | 0.03 |
| Temperament*Angry facial expressions | -0.0003 | 0.02 | -0.01 | 448.77 | 0.99 |
| Temperament*Sad facial expressions | -0.02 | 0.02 | -0.99 | 1046.10 | 0.32 |
| *Step 3* |  |  |  |  |  |
| Intercept | 0.84 | 0.08 | 10.00 | 811.37 | <0.001 |
| Temperament | 0.09 | 0.02 | 4.24 | 2668.10 | <0.001 |
| Angry facial expressions | 0.0002 | 0.02 | 0.01 | 373.44 | 0.99 |
| Sad facial expressions | -0.05 | 0.02 | -2.13 | 748.48 | 0.03 |
| Sex: girl vs boy | 0.09 | 0.05 | 1.65 | 1512.64 | 0.10 |
| Age at CBCL | 0.02 | 0.02 | 1.15 | 2653.14 | 0.25 |
| Puberty status at CBCL | 0.0001 | 0.04 | 0.003 | 543.76 | 1.00 |
| Child national origin: non-Dutch vs Dutch | -0.04 | 0.05 | -0.83 | 2612.17 | 0.41 |
| Maternal age | 0.07 | 0.02 | 3.44 | 2680.88 | <0.001 |
| Maternal education: other vs university | -0.04 | 0.05 | -0.74 | 2190.67 | 0.46 |
| Temperament*Angry facial expressions | -0.004 | 0.02 | -0.17 | 492.28 | 0.87 |
| Temperament*Sad facial expressions | -0.02 | 0.02 | -1.05 | 990.97 | 0.29 |

Note: CBCL = Child Behavior Checklist.

**Appendix S3. Observed temperament**

Even though the association between observed temperament at 3 years and anxiety symptoms at 13 years was not significant, β=0.01, *p*=0.77 (Table S3a), we repeated the analyses with possible moderators with observed temperament instead of parent-reported temperament as independent variable.

The analyses with victimization yielded similar results as the main analysis: children who were victimized more showed more anxiety symptoms, β=0.15, *p*=0.01, but there was no interaction between observed temperament and victimization, β=0.08, *p*=0.15. For friendship quality, there was only an interaction with observed temperament, β=0.11, *p*=0.04. For negative feelings, there was only a main effect of negative feelings after social exclusion, β=0.11, *p*=0.03, no interaction with observed temperament, β=-0.003, *p*=0.96 (Table S3b). There were no significant main effects of nor interactions with angry and sad facial expressions.

Table S3a. Results of the robust linear regression model with observed temperament as independent variable and anxiety as dependent variable.

|  | *beta* | *SE* | *t* | *df* | *p* |
| --- | --- | --- | --- | --- | --- |
| *Step 1* |  |  |  |  |  |
| Intercept | 0.85 | 0.06 | 13.86 | 637.44 | <0.001 |
| Temperament | 0.01 | 0.04 | 0.29 | 637.90 | 0.77 |
| Sex: girl vs boy | 0.04 | 0.09 | 0.48 | 637.71 | 0.63 |
| *Step 2* |  |  |  |  |  |
| Intercept | 0.85 | 0.06 | 13.84 | 636.46 | <0.001 |
| Temperament | -0.02 | 0.06 | -0.29 | 636.94 | 0.77 |
| Sex: girl vs boy | 0.04 | 0.09 | 0.48 | 636.71 | 0.63 |
| Temperament*Sex | 0.07 | 0.09 | 0.76 | 636.88 | 0.45 |
| *Step 3* |  |  |  |  |  |
| Intercept | 0.85 | 0.17 | 5.09 | 469.54 | <0.001 |
| Temperament | -0.01 | 0.06 | -0.16 | 632.79 | 0.88 |
| Sex: girl vs boy | 0.01 | 0.11 | 0.14 | 597.50 | 0.89 |
| Temperament*Sex | 0.06 | 0.09 | 0.60 | 632.81 | 0.55 |
| Age (in years) at CBCL | -0.07 | 0.04 | -1.64 | 629.93 | 0.10 |
| Puberty status at CBCL | 0.03 | 0.08 | 0.34 | 418.34 | 0.73 |
| Maternal age | 0.05 | 0.04 | 1.02 | 631.50 | 0.31 |
| Maternal education: other vs university | -0.06 | 0.09 | -0.58 | 615.89 | 0.56 |

Note: CBCL = Child Behavior Checklist

Table S3b. Results of the robust linear regression model with anxiety as dependent variable and negative feelings as moderator, with observed temperament.

|  | *beta* | *SE* | *t* | df | *p* |
| --- | --- | --- | --- | --- | --- |
| *Step 1* |  |  |  |  |  |
| Intercept | 0.86 | 0.06 | 13.91 | 632.09 | <0.001 |
| Temperament | 0.01 | 0.04 | 0.22 | 634.61 | 0.82 |
| Negative feelings during Cyberball | 0.04 | 0.05 | 0.87 | 404.84 | 0.39 |
| Negative feelings after Cyberball | 0.11 | 0.05 | 2.19 | 321.32 | 0.03 |
| Sex | 0.03 | 0.09 | 0.37 | 631.49 | 0.71 |
| *Step 2* |  |  |  |  |  |
| Intercept | 0.86 | 0.06 | 13.94 | 629.68 | <0.001 |
| Temperament | 0.01 | 0.04 | 0.26 | 625.91 | 0.79 |
| Negative feelings during Cyberball | 0.05 | 0.05 | 0.95 | 389.92 | 0.34 |
| Negative feelings after Cyberball | 0.10 | 0.05 | 2.06 | 317.51 | 0.04 |
| Sex | 0.03 | 0.09 | 0.29 | 628.02 | 0.77 |
| Temperament*Negative feelings during Cyberball | -0.09 | 0.05 | -1.84 | 445.37 | 0.07 |
| Temperament*Negative feelings after Cyberball | -0.003 | 0.05 | -0.05 | 321.60 | 0.96 |
| *Step 3* |  |  |  |  |  |
| Intercept | 0.87 | 0.17 | 5.21 | 483.59 | <0.001 |
| Temperament | 0.01 | 0.05 | 0.30 | 621.12 | 0.76 |
| Negative feelings during Cyberball | 0.04 | 0.05 | 0.83 | 385.06 | 0.41 |
| Negative feelings after Cyberball | 0.12 | 0.05 | 2.37 | 365.50 | 0.02 |
| Sex: girl vs boy | -0.004 | 0.11 | -0.03 | 581.71 | 0.97 |
| Age at CBCL | -0.08 | 0.04 | -1.91 | 621.39 | 0.06 |
| Puberty status at CBCL | 0.02 | 0.08 | 0.32 | 426.78 | 0.75 |
| Maternal age | 0.05 | 0.04 | 1.08 | 626.19 | 0.28 |
| Maternal education: other vs university | -0.06 | 0.10 | -0.68 | 606.78 | 0.50 |
| Temperament*Negative feelings during Cyberball | -0.09 | 0.05 | -1.81 | 450.11 | 0.07 |
| Temperament*Negative feelings after Cyberball | -0.002 | 0.05 | -0.03 | 314.26 | 0.97 |

Note: CBCL = Child Behavior Checklist.

**Appendix S4. Peer-reported victimization and friendships**

Peer-reported data on victimization and friendships were administered at school with a computerized peer-nomination instrument (peer evaluation of relationships at school [PEERS] Measure; (Verlinden et al., 2014)) in a subset of children when they were 7 years. Peer-reported data on victimization was collected for 615 children (328 girls) out of the 2730 children with temperament and anxiety data, and peer-reported data on friendships was collected for 583 children (313 girls) out of the 2730 children with temperament and anxiety data. Missing data was imputed using the mice package (van Buuren & Groothuis-Oudshoorn, 2011) with a maximum of 100 iterations creating 30 datasets (same as in main analysis).

There was no interaction between temperament and peer-reported victimization, *b*=0.04, *p*=0.49, or peer-reported friendships, *b*=-0.01, *p*=0.75, in predicting anxiety symptoms (Table S4).

Table S4. Results of the robust linear regression model with anxiety as dependent variable and peer-reported victimization and friendships as moderators.

|  | *beta* | *SE* | *t* | df | *p* |
| --- | --- | --- | --- | --- | --- |
| Step 1 |  |  |  |  |  |
| Intercept | 0.77 | 0.03 | 25.79 | 2566.87 | <0.001 |
| Temperament | 0.07 | 0.02 | 3.33 | 2426.38 | <0.001 |
| Peer-reported victimization | 0.06 | 0.04 | 1.50 | 50.16 | 0.14 |
| Peer-reported friendships | -0.05 | 0.04 | -1.28 | 47.64 | 0.21 |
| Sex: girl vs boy | 0.09 | 0.04 | 2.20 | 2511.29 | 0.03 |
| Step 2 |  |  |  |  |  |
| Intercept | 0.77 | 0.03 | 25.60 | 2372.32 | <0.001 |
| Temperament | 0.07 | 0.02 | 3.28 | 2034.93 | 0.001 |
| Peer-reported victimization | 0.05 | 0.04 | 1.45 | 51.97 | 0.15 |
| Peer-reported friendships | -0.05 | 0.04 | -1.27 | 48.47 | 0.21 |
| Sex: girl vs boy | 0.09 | 0.04 | 2.19 | 2449.52 | 0.03 |
| Temperament*Peer-reported victimization | 0.02 | 0.04 | 0.69 | 50.98 | 0.49 |
| Temperament*Peer-reported friendships | -0.01 | 0.03 | -0.32 | 69.41 | 0.75 |
| Step 3 |  |  |  |  |  |
| Intercept | 0.88 | 0.09 | 9.94 | 462.58 | <0.001 |
| Temperament | 0.09 | 0.02 | 4.18 | 2417.56 | <0.001 |
| Peer-reported victimization | 0.07 | 0.04 | 1.82 | 52.86 | 0.07 |
| Peer-reported friendships | -0.06 | 0.04 | -1.48 | 48.52 | 0.15 |
| Sex: girl vs boy | 0.10 | 0.05 | 1.79 | 1020.29 | 0.07 |
| Age at CBCL | 0.03 | 0.02 | 1.27 | 2479.32 | 0.20 |
| Puberty status at CBCL | -0.01 | 0.04 | -0.27 | 381.33 | 0.79 |
| Child national origin: non-Dutch vs Dutch | -0.06 | 0.05 | -1.32 | 2043.51 | 0.19 |
| Maternal age | 0.07 | 0.02 | 3.57 | 2338.60 | <0.001 |
| Maternal education: other vs university | -0.06 | 0.05 | -1.15 | 1690.43 | 0.25 |

Note: CBCL = Child Behavior Checklist.

**Appendix S5. Self-reported anxiety symptoms**

Participants also filled out the Youth Self-Report (Achenbach, 1991) questionnaire as a measure of self-reported anxiety symptoms when they were 13 years. Like for the CBCL, we used the DSM anxiety scale (Achenbach et al., 2003; Ebesutani et al., 2010; Nakamura et al., 2009). 2619 children (1361 girls) have data on parent-reported temperament and self-reported anxiety symptoms. All were similar as to the analyses described in the paper.

As in the main analysis, children with higher scores on parent-reported fearful temperament at 6 months showed more anxiety symptoms when they were 13 years, β=0.10, *p*<0.001. Also, the analyses with victimization, friendship quality, and negative facial expressions as moderator (3 sets of analyses) yielded similar results: main effects of victimization, friendship quality, and sad facial expressions, but no interactions with temperament. However, the interaction between temperament and negative feelings after social exclusion was no longer significant when outliers were excluded, *b*=0.06, *p*=0.12 (Table S5).

Table S5. Results of the robust linear regression model with self-reported anxiety as dependent variable and negative feelings as moderator.

|  | *beta* | *SE* | *t* | df | *p* |
| --- | --- | --- | --- | --- | --- |
| *Step 1* |  |  |  |  |  |
| Intercept | 1.60 | 0.04 | 35.75 | 2510.35 | <0.001 |
| Temperament | 0.11 | 0.03 | 3.64 | 2532.55 | <0.001 |
| Negative feelings during Cyberball | 0.09 | 0.04 | 2.41 | 638.68 | 0.02 |
| Negative feelings after Cyberball | 0.28 | 0.04 | 8.02 | 462.09 | <0.001 |
| Sex | 0.57 | 0.06 | 8.74 | 2437.66 | <0.001 |
| *Step 2* |  |  |  |  |  |
| Intercept | 1.60 | 0.04 | 35.55 | 2466.26 | <0.001 |
| Temperament | 0.11 | 0.03 | 3.52 | 2164.17 | <0.001 |
| Negative feelings during Cyberball | 0.09 | 0.04 | 2.45 | 616.96 | 0.01 |
| Negative feelings after Cyberball | 0.28 | 0.04 | 7.89 | 526.30 | <0.001 |
| Sex | 0.57 | 0.06 | 8.72 | 2396.78 | <0.001 |
| Temperament*Negative feelings during Cyberball | -0.03 | 0.04 | -0.81 | 159.59 | 0.42 |
| Temperament*Negative feelings after Cyberball | 0.06 | 0.04 | 1.57 | 208.63 | 0.12 |
| *Step 3* |  |  |  |  |  |
| Intercept | 1.76 | 0.12 | 14.25 | 1107.88 | <0.001 |
| Temperament | 0.12 | 0.03 | 3.92 | 2326.54 | <0.001 |
| Negative feelings during Cyberball | 0.08 | 0.04 | 2.21 | 601.65 | 0.03 |
| Negative feelings after Cyberball | 0.28 | 0.04 | 7.88 | 538.12 | <0.001 |
| Sex: girl vs boy | 0.58 | 0.08 | 7.35 | 1881.40 | <0.001 |
| Age at YSR | 0.05 | 0.03 | 1.63 | 2323.14 | 0.10 |
| Puberty status at YSR | -0.02 | 0.06 | -0.42 | 883.03 | 0.68 |
| Child national origin: non-Dutch vs Dutch | -0.17 | 0.07 | -2.48 | 2516.93 | 0.01 |
| Maternal age | -0.02 | 0.03 | -0.78 | 2430.98 | 0.43 |
| Maternal education: other vs university | -0.11 | 0.07 | -1.53 | 2272.92 | 0.13 |
| Temperament*Negative feelings during Cyberball | -0.03 | 0.04 | -0.89 | 169.71 | 0.38 |
| Temperament*Negative feelings after Cyberball | 0.05 | 0.04 | 1.51 | 223.79 | 0.13 |

Note: CBCL = Child Behavior Checklist.

**Appendix S6. All peer interaction variables in one model**

Table S6. Results of the first sensitivity analysis including all peer interaction variables.

|  | *beta* | *SE* | *t* | df | *p* |
| --- | --- | --- | --- | --- | --- |
| *Step 1* |  |  |  |  |  |
| Intercept | 0.78 | 0.03 | 25.76 | 2422.64 | <0.001 |
| Temperament | 0.08 | 0.02 | 3.80 | 2612.64 | <0.001 |
| Victimization | 0.15 | 0.03 | 5.01 | 96.00 | <0.001 |
| Friendship Quality | -0.03 | 0.02 | -1.37 | 410.62 | 0.17 |
| Negative feelings during Cyberball | 0.01 | 0.02 | 0.38 | 451.48 | 0.71 |
| Negative feelings after Cyberball | 0.13 | 0.03 | 5.02 | 280.32 | <0.001 |
| Angry facial expressions | -0.003 | 0.02 | -0.14 | 296.57 | 0.89 |
| Sad facial expressions | -0.06 | 0.02 | -2.43 | 867.09 | 0.02 |
| Sex: girl vs boy | 0.10 | 0.04 | 2.29 | 2341.51 | 0.02 |
| *Step 2* |  |  |  |  |  |
| Intercept | 0.78 | 0.03 | 25.80 | 2327.45 | <0.001 |
| Temperament | 0.09 | 0.02 | 4.40 | 1162.10 | <0.001 |
| Victimization | 0.15 | 0.03 | 5.10 | 111.22 | <0.001 |
| Friendship Quality | -0.03 | 0.02 | -1.34 | 473.56 | 0.18 |
| Negative feelings during Cyberball | 0.01 | 0.02 | 0.33 | 504.22 | 0.74 |
| Negative feelings after Cyberball | 0.12 | 0.03 | 4.96 | 304.05 | <0.001 |
| Angry facial expressions | -0.004 | 0.02 | -0.15 | 284.02 | 0.88 |
| Sad facial expressions | -0.05 | 0.02 | -2.29 | 1007.02 | 0.02 |
| Sex: girl vs boy | 0.10 | 0.04 | 2.25 | 2267.52 | 0.02 |
| Temperament*Victimization | 0.03 | 0.03 | 0.93 | 78.50 | 0.36 |
| Temperament*Friendship quality | 0.003 | 0.03 | 0.14 | 181.57 | 0.89 |
| Temperament*Negative feelings during Cyberball | 0.02 | 0.03 | 0.64 | 223.33 | 0.53 |
| Temperament*Negative feelings after Cyberball | 0.05 | 0.02 | 2.13 | 177.37 | 0.03 |
| Temperament*Angry facial expressions | 0.002 | 0.02 | 0.10 | 386.83 | 0.92 |
| Temperament*Sad facial expressions | -0.03 | 0.02 | -1.26 | 652.14 | 0.21 |
| *Step 3* |  |  |  |  |  |
| Intercept | 0.85 | 0.09 | 9.90 | 574.55 | <0.001 |
| Temperament | 0.11 | 0.02 | 5.00 | 1439.33 | <0.001 |
| Victimization | 0.15 | 0.03 | 5.12 | 120.92 | <0.001 |
| Friendship Quality | -0.03 | 0.02 | -1.25 | 546.99 | 0.21 |
| Negative feelings during Cyberball | 0.002 | 0.02 | 0.10 | 551.64 | 0.92 |
| Negative feelings after Cyberball | 0.12 | 0.02 | 5.02 | 357.90 | <0.001 |
| Angry facial expressions | -0.002 | 0.02 | -0.09 | 276.74 | 0.93 |
| Sad facial expressions | -0.05 | 0.02 | -2.15 | 939.36 | 0.03 |
| Sex: girl vs boy | 0.10 | 0.05 | 1.85 | 1485.57 | 0.06 |
| Age at CBCL | 0.04 | 0.02 | 1.77 | 2490.74 | 0.08 |
| Puberty status at CBCL | -0.01 | 0.04 | -0.18 | 428.67 | 0.86 |
| Child national origin: non-Dutch vs Dutch | -0.02 | 0.05 | -0.41 | 2459.90 | 0.68 |
| Maternal age | 0.07 | 0.02 | 3.41 | 2335.88 | <0.001 |
| Maternal education: other vs university | -0.03 | 0.05 | -0.72 | 1962.41 | 0.47 |
| Temperament*Victimization | 0.03 | 0.03 | 0.95 | 80.22 | 0.35 |
| Temperament*Friendship quality | 0.001 | 0.03 | 0.05 | 187.33 | 0.96 |
| Temperament*Negative feelings during Cyberball | 0.02 | 0.02 | 0.77 | 252.10 | 0.44 |
| Temperament*Negative feelings after Cyberball | 0.05 | 0.02 | 2.21 | 191.98 | 0.03 |
| Temperament*Angry facial expressions | -0.002 | 0.02 | -0.11 | 410.06 | 0.91 |
| Temperament*Sad facial expressions | -0.03 | 0.02 | -1.35 | 661.76 | 0.18 |

Note: CBCL = Child Behavior Checklist.

**Appendix S7. Without outliers**

We removed outliers (defined as values that are 3 SDs above/below the mean) for temperament and the moderators. Table S7a shows the percentage of outliers per variable. We then imputed all missing variables using the mice package (van Buuren & Groothuis-Oudshoorn, 2011) with a maximum of 100 iterations creating 30 datasets (same as in main analysis).

As in the main analysis, children with higher scores on parent-reported fearful temperament at 6 months showed more anxiety symptoms when they were 13 years, β=0.07, *p*=0.001. Also, the analyses with victimization, friendship quality, and negative facial expressions as moderator (3 sets of analyses) yielded similar results: main effects of victimization, friendship quality, and sad facial expressions, but no interactions with temperament. However, the interaction between temperament and negative feelings after social exclusion was no longer significant when outliers were excluded, *b*=0.02, *p*=0.48 (Table S7b).

Table S7a. Percentage of outliers per variable.

|  | % outliers |
| --- | --- |
| Parent-reported temperament | 0.81 |
| Victimization | 1.83 |
| Friendship quality | 0.22 |
| Negative feelings during Cyberball | 0 |
| Negative feelings after Cyberball | 0.81 |
| Angry facial expressions | 1.94 |
| Sad facial expressions | 2.09 |

Table S7b. Results of the robust linear regression model with anxiety as dependent variable and negative feelings as moderator, excluding outliers.

|  | *beta* | *SE* | *t* | df | *p* |
| --- | --- | --- | --- | --- | --- |
| *Step 1* |  |  |  |  |  |
| Intercept | 0.78 | 0.03 | 26.21 | 2624.89 | <0.001 |
| Temperament | 0.07 | 0.02 | 3.25 | 2373.94 | 0.001 |
| Negative feelings during Cyberball | 0.01 | 0.02 | 0.23 | 485.04 | 0.82 |
| Negative feelings after Cyberball | 0.13 | 0.02 | 5.25 | 357.41 | <0.001 |
| Sex | 0.10 | 0.04 | 2.26 | 2627.05 | 0.02 |
| *Step 2* |  |  |  |  |  |
| Intercept | 0.78 | 0.03 | 26.26 | 2645.11 | <0.001 |
| Temperament | 0.07 | 0.02 | 3.41 | 1995.36 | <0.001 |
| Negative feelings during Cyberball | 0.01 | 0.02 | 0.24 | 509.52 | 0.81 |
| Negative feelings after Cyberball | 0.13 | 0.02 | 5.20 | 418.86 | <0.001 |
| Sex | 0.10 | 0.04 | 2.28 | 2622.59 | 0.02 |
| Temperament*Negative feelings during Cyberball | 0.02 | 0.02 | 0.70 | 296.98 | 0.48 |
| Temperament*Negative feelings after Cyberball | 0.02 | 0.03 | 0.94 | 135.96 | 0.35 |
| *Step 3* |  |  |  |  |  |
| Intercept | 0.84 | 0.08 | 9.93 | 772.85 | <0.001 |
| Temperament | 0.09 | 0.02 | 4.03 | 2098.26 | <0.001 |
| Negative feelings during Cyberball | -0.001 | 0.02 | -0.02 | 540.65 | 0.98 |
| Negative feelings after Cyberball | 0.13 | 0.02 | 5.15 | 415.16 | <0.001 |
| Sex: girl vs boy | 0.09 | 0.05 | 1.76 | 1739.49 | 0.08 |
| Age at CBCL | 0.03 | 0.02 | 1.55 | 2595.49 | 0.12 |
| Puberty status at CBCL | -0.002 | 0.04 | -0.04 | 558.61 | 0.96 |
| Child national origin: non-Dutch vs Dutch | -0.03 | 0.05 | -0.77 | 2644.62 | 0.44 |
| Maternal age | 0.07 | 0.02 | 3.41 | 2600.16 | <0.001 |
| Maternal education: other vs university | -0.02 | 0.05 | -0.50 | 2117.92 | 0.62 |
| Temperament*Negative feelings during Cyberball | 0.02 | 0.02 | 0.80 | 301.84 | 0.42 |
| Temperament*Negative feelings after Cyberball | 0.03 | 0.03 | 1.03 | 141.71 | 0.30 |

Note: CBCL = Child Behavior Checklist.

**Appendix S8. Depressive symptoms as additional covariate**

Depressive symptoms were also measured with the Child Behavior Checklist (Achenbach & Rescorla, 2001), using the DSM affective disorders subscale (Achenbach et al., 2003; Ebesutani et al., 2010; Nakamura et al., 2009). Self-reported data on depressive symptoms were collected for 2729 children (1393 girls) out of the 2730 children with temperament and anxiety data. Missing data was imputed using the mice package (van Buuren & Groothuis-Oudshoorn, 2011) with a maximum of 100 iterations creating 30 datasets (same as in main analysis).

All analyses showed a strong effect of depressive symptoms: children with more depressive symptoms also showed more anxiety symptoms, βs>0.85, *p*s<0.001. As in the main analysis, children with higher scores on parent-reported fearful temperament at 6 months showed more anxiety symptoms when they were 13 years, β=0.08, *p*=0.001. Also, the analyses with victimization and negative facial expressions as moderator (3 sets of analyses) yielded similar results: main effects of victimization and sad facial expressions, but no interactions with temperament. However, the main effect of friendship quality and the interaction between temperament and negative feelings after social exclusion were no longer significant when depressive symptoms were added as covariate, respectively *b*=-0.02, *p*=0.37 and *b*=0.01, *p*=0.55 (Table S8).

Table S8. Results of the robust linear regression model with anxiety as dependent variable and negative feelings as moderator, depressive symptoms as additional covariate.

|  | *beta* | *SE* | *t* | df | *p* |
| --- | --- | --- | --- | --- | --- |
| Analysis 1 |  |  |  |  |  |
| Intercept | 1.02 | 0.08 | 13.24 | 338.77 | <0.001 |
| Temperament | 0.06 | 0.02 | 3.24 | 2093.13 | 0.001 |
| Victimization | 0.07 | 0.02 | 3.11 | 169.04 | 0.002 |
| Sex: girl vs boy | 0.17 | 0.05 | 3.75 | 1006.24 | <0.001 |
| Depressive symptoms | 0.85 | 0.02 | 47.03 | 2178.26 | <0.001 |
| Age at CBCL | 0.01 | 0.02 | 0.49 | 2585.93 | 0.63 |
| Puberty status at CBCL | -0.06 | 0.04 | -1.56 | 223.17 | 0.12 |
| Child national origin: non-Dutch vs Dutch | -0.02 | 0.04 | -0.50 | 2581.25 | 0.61 |
| Maternal age | 0.05 | 0.02 | 2.72 | 2615.18 | 0.01 |
| Maternal education: other vs university | -0.04 | 0.04 | -1.02 | 1906.38 | 0.31 |
| Temperament*Victimization | 0.04 | 0.02 | 1.92 | 148.81 | 0.06 |
| Analysis 2 |  |  |  |  |  |
| Intercept | 1.01 | 0.08 | 13.27 | 375.63 | <0.001 |
| Temperament | 0.05 | 0.02 | 2.81 | 2692.32 | 0.01 |
| Friendship quality | -0.02 | 0.02 | -0.89 | 563.11 | 0.37 |
| Sex: girl vs boy | 0.18 | 0.05 | 3.90 | 1086.73 | <0.001 |
| Depressive symptoms | 0.86 | 0.02 | 48.97 | 2598.51 | <0.001 |
| Age at CBCL | 0.01 | 0.02 | 0.34 | 2630.22 | 0.73 |
| Puberty status at CBCL | -0.06 | 0.04 | -1.57 | 246.33 | 0.12 |
| Child national origin: non-Dutch vs Dutch | -0.03 | 0.04 | -0.82 | 2622.65 | 0.41 |
| Maternal age | 0.04 | 0.02 | 2.55 | 2647.10 | 0.01 |
| Maternal education: other vs university | -0.03 | 0.04 | -0.85 | 2003.21 | 0.39 |
| Temperament*Friendship quality | -0.01 | 0.02 | -0.54 | 320.95 | 0.59 |
| Analysis 3 |  |  |  |  |  |
| Intercept | 1.02 | 0.08 | 13.31 | 366.79 | <0.001 |
| Temperament | 0.05 | 0.02 | 3.00 | 2576.53 | 0.003 |
| Negative feelings during Cyberball | -0.04 | 0.02 | -1.68 | 489.44 | 0.09 |
| Negative feelings after Cyberball | 0.04 | 0.02 | 2.04 | 345.24 | 0.04 |
| Sex: girl vs boy | 0.16 | 0.05 | 3.50 | 962.40 | <0.001 |
| Depressive symptoms | 0.86 | 0.02 | 47.88 | 2567.15 | <0.001 |
| Age at CBCL | 0.01 | 0.02 | 0.43 | 2571.59 | 0.67 |
| Puberty status at CBCL | -0.05 | 0.04 | -1.48 | 242.75 | 0.14 |
| Child national origin: non-Dutch vs Dutch | -0.03 | 0.04 | -0.81 | 2644.41 | 0.42 |
| Maternal age | 0.05 | 0.02 | 2.74 | 2622.59 | 0.01 |
| Maternal education: other vs university | -0.04 | 0.04 | -0.89 | 1955.25 | 0.38 |
| Temperament*Negative feelings during Cyberball | 0.02 | 0.02 | 1.05 | 557.01 | 0.30 |
| Temperament*Negative feelings after Cyberball | 0.01 | 0.02 | 0.60 | 246.04 | 0.55 |
| Analysis 4 |  |  |  |  |  |
| Intercept | 1.01 | 0.08 | 13.27 | 363.39 | <0.001 |
| Temperament | 0.05 | 0.02 | 2.88 | 2643.68 | 0.004 |
| Angry facial expressions | -0.02 | 0.02 | -1.05 | 990.33 | 0.30 |
| Sad facial expressions | -0.04 | 0.02 | -2.23 | 1135.66 | 0.03 |
| Sex: girl vs boy | 0.17 | 0.05 | 3.70 | 1074.59 | <0.001 |
| Depressive symptoms | 0.86 | 0.02 | 49.12 | 2609.04 | <0.001 |
| Age at CBCL | 0.004 | 0.02 | 0.24 | 2622.53 | 0.81 |
| Puberty status at CBCL | -0.05 | 0.04 | -1.55 | 246.96 | 0.12 |
| Child national origin: non-Dutch vs Dutch | -0.02 | 0.04 | -0.60 | 2592.05 | 0.55 |
| Maternal age | 0.05 | 0.02 | 2.64 | 2570.42 | 0.01 |
| Maternal education: other vs university | -0.03 | 0.04 | -0.76 | 1916.10 | 0.45 |
| Temperament*Angry facial expressions | 0.01 | 0.02 | 0.37 | 308.05 | 0.71 |
| Temperament*Sad facial expressions | -0.02 | 0.02 | -0.95 | 895.89 | 0.34 |

Note: CBCL = Child Behavior Checklist.

**References**

Achenbach, T. M. (1991). *Manual for the youth self-report and 1991 profile*. University of Vermont.

Achenbach, T. M., Dumenci, L., & Rescorla, L. A. (2003). DSM-oriented and empirically based approaches to constructing scales from the same item pools. *Journal of Clinical Child and Adolescent Psychology*, *32*(3), 328-340. <https://doi.org/10.1207/s15374424jccp3203_02>

Achenbach, T. M., & Rescorla, L. A. (2001). *Manual for the ASEBA School-Age Forms & Profiles*. University of Vermont, Research Center for Children, Youth and Families.

Ebesutani, C., Bernstein, A., Nakamura, B. J., Chorpita, B. F., Higa-McMillan, C. K., Weisz, J. R., & Res Network Youth Mental, H. (2010). Concurrent validity of the Child Behavior Checklist DSM-oriented scales: Correspondence with DSM diagnoses and comparison to syndrome scales [Article]. *Journal of Psychopathology and Behavioral Assessment*, *32*(3), 373-384. <https://doi.org/10.1007/s10862-009-9174-9>

Nakamura, B. J., Ebesutani, C., Bernstein, A., & Chorpita, B. F. (2009). A psychometric analysis of the Child Behavior Checklist DSM-oriented scales [Article]. *Journal of Psychopathology and Behavioral Assessment*, *31*(3), 178-189. <https://doi.org/10.1007/s10862-008-9119-8>

van Buuren, S., & Groothuis-Oudshoorn, K. (2011). mice: Multivariate Imputation by Chained Equations in R [Article]. *Journal of Statistical Software*, *45*(3), 1-67.

Verlinden, M., Veenstra, R., Ringoot, A. P., Jansen, P. W., Raat, H., Hofman, A., . . . Tiemeier, H. (2014). Detecting bullying in early elementary school with a computerized peer-nomination instrument. *Psychological Assessment*, *26*(2), 628-641. <https://doi.org/10.1037/a0035571>
